# Supplementary material for: Comparison of Characteristics and Survival Rates of Resectable Pancreatic Ductal Adenocarcinoma according to Tumor Location
Source: Biomedicines. 2021 Nov 17;9(11):1706. doi: 10.3390/biomedicines9111706 (PMC8615679; doi:10.3390/biomedicines9111706)
Supplement: Supplementary file 1 [file biomedicines-09-01706-s001.zip › biomedicines-1446277-supplementary.pdf]

Supplementary material

Table S1. Multivariate analysis for predictive factors of overall survival of pancreatic head cancer patients

		Univariate		Multivariate	
		HR (95% CI)		HR (95% CI)	
Age (yrs)	< 65	1.000		1.000	
	≥ 65	1.183 (1.058 – 1.324)	0.003	1.134 (1.007 – 1.276)	0.037
Sex	Male	1.000			
	Female	1.058 (0.945 – 1.185)	0.328		
BMI (kg/m^2)	< 25	1.000			
	≥ 25	0.846 (0.733 – 0.976)	0.022		
CA 19-9	Normal	1.000			
	Increased	1.243 (1.100 - 1.406)	0.001		
CEA	Normal	1.000			
	Increased	1.135 (0.978 – 1.317)	0.096		
mGPS	0	1.000		1.000	
	1	1.435 (1.146– 1.797)	0.002	1.490 (1.186 – 1.872)	0.001
	2	1.208 (1.023 - 1.427)	0.026	1.047 (0.883 – 1.241)	0.600
Period	2000 – 2009	1.000			
	2010 – 2018	0.696 (0.620 – 0.782)	0.001		
Length of hospital stay	≤ 15	1.000		1.000	
	> 15	1.374 (1.227 – 1.538)	0.001	1.304 (1.157 – 1.471)	0.001
POPF	No	1.000		1.000	
	Grade A	0.866 (0.705 - 1.064)	0.170	0.820 (0.661 – 1.018)	0.072
	Grade B or C	0.893 (0.665 - 1.200)	0.453	0.676 (0.493 – 0.926)	0.015
Complications	No	1.000		1.000	
	Grade I-II	1.140 (1.009 – 1.289)	0.035	1.201 (1.050 – 1.373)	0.007
	Grade III-IV	1.057 (0.841 - 1.328)	0.635	1.061 (0.883 – 1.350)	0.633
Adjuvant therapy	No	1.000		1.000	
	CTx	0.714 (0.630 – 0.809)	0.001	0.702 (0.616 – 0.799)	0.001
	CCRTx	0.711 (0.598 – 0.844)	0.001	0.633 (0.529 – 0.757)	0.001
Operation method	Open	1.000			
	Lap/robot	0.638 (0.462 – 0.882)	0.006		
Vein resection	No	1.000		1.000	
	Yes	1.494 (1.332 – 1.677)	0.001	1.247 (1.105 – 1.407)	0.001
Artery resection	No	1.000			
	Yes	1.420 (1.121 – 1.799)	0.004		
Combined resection	No	1.000			
	Yes	1.500 (1.044 – 2.155)	0.028		
Tumor size (cm)		1.179 (1.137 – 1.223)	0.001	1.143 (1.092 – 1.196)	0.001
Differentiation	Well	1.000		1.000	
	Moderate	1.573 (1.286 – 1.925)	0.001	1.451 (1.189 – 1.771)	0.001
	Poor	2.839 (2.233 – 3.610)	0.001	2.545 (2.004 – 3.231)	0.001
T stage	T1	1.000			
	T2	1.550 (1.294 – 1.857)	0.001		
	T3	2.104 (1.694 – 2.615)	0.001		
	T4	2.442 (1.474 – 4.045)	0.001		
N stage	N0	1.000			
	N1	1.682 (1.482 – 1.910)	0.001		

	N2	2.411 (2.053 – 2.830)	0.001		
M stage	M0	1.000			
	M1	1.867 (1.398 – 2.492)	0.001		
TNM stage	IA	1.000		1.000	
	IB	1.517 (1.154 - 1.994)	0.001	1.188 (0.898 – 1.572)	0.228
	IIA	1.790 (1.244 – 2.574)	0.001	1.073 (0.714 – 1.613)	0.734
	IIB	2.399 (1.849 – 3.113)	0.001	1.759 (1.337 – 2.312)	0.001
	III	3.397 (2.569 – 4.494)	0.001	2.355 (1.752 – 3.166)	0.001
	IV	3.887 (2.667 – 5.664)	0.001	2.771 (1.874 – 4.097)	0.001
LVI	No	1.000		1.000	
	Yes	1.459 (1.303 – 1.634)	0.001	1.314 (1.166 – 1.481)	0.001
PNI	No	1.000			
	Yes	1.333 (1.140 – 1.560)	0.001		
RM	R0	1.00			
	R1	1.289 (1.135 – 1.465)	0.001		

BMI, body mass index; CA, carbohydrate antigen; CEA, carcinoembryonic antigen; mGPS, modified Glasgow prognostic score; POPF, postoperative pancreatic fistula; CTx, chemotherapy; CCRTx, concurrent chemoradiation therapy; LVI, lymphovascular invasion; PNI, perineural invasion; RM, resection margin

Table S2. Multivariate analysis for predictive factors of overall survival of pancreatic body or tail cancer patients

		Univariate		Multivariate	
		HR (95% CI)		HR (95% CI)	
Age (yrs)	< 65	1.000			
	≥ 65	0.918 (0.776 – 1.087)	0.320		
Sex	Male	1.000			
	Female	1.264 (1.065 – 1.500)	0.007		
BMI (kg/m²)	< 25	1.000		1.000	
	≥ 25	0.751 (0.619 – 0.911)	0.004	0.816 (0.669 – 0.994)	0.044
CA 19-9	Normal	1.000		1.000	
	Increased	1.661 (1.388 - 1.988)	0.001	1.483 (1.233 – 1.783)	0.001
CEA	Normal	1.000			
	Increased	1.344 (1.076 – 1.678)	0.009		
mGPS	0	1.000			
	1	0.863 (0.577– 1.291)	0.474		
	2	1.504 (1.085 – 2.084)	0.014		
Period	2000 – 2009	1.000			
	2010 – 2018	0.605 (0.507 – 0.722)	0.001		
Length of hospital stay	≤ 15	1.000		1.000	
	> 15	1.599 (1.349 – 1.895)	0.001	1.325 (1.108 – 1.585)	0.002
POPF	No	1.000			
	Grade A	0.983 (0.801 - 1.206)	0.867		
	Grade B or C	1.170 (0.867- 1.580)	0.304		
Complications	No	1.000			
	Grade I-II	1.258 (1.044 – 1.516)	0.016		
	Grade III-IV	1.267 (0.937 - 1.715)	0.125		
Adjuvant therapy	No	1.000		1.000	
	CTx	0.793 (0.652 – 0.964)	0.020	0.696 (0.568 – 0.853)	0.001
	CCRTx	1.063 (0.833 – 1.356)	0.623	0.731 (0.565 – 0.946)	0.017
Operation method	Open	1.000		1.000	
	Lap/robot	0.584 (0.490 – 0.698)	0.001	0.694 (0.575 – 0.837)	0.001
Vein resection	No	1.000			
	Yes	1.669 (1.304 – 2.136)	0.001		
Artery resection	No	1.000			
	Yes	2.004 (1.466 – 2.739)	0.001		
Combined resection	No	1.000			
	Yes	1.148 (0.889 – 1.483)	0.289		
Tumor size (cm)		1.197 (1.154 – 1.242)	0.001	1.093 (1.040 – 1.148)	0.001
Differentiation	Well	1.000		1.000	
	Moderate	1.903 (1.418 – 2.554)	0.001	1.460 (1.084 – 1.967)	0.013
	Poor	2.836 (1.988 – 4.046)	0.001	2.140 (1.494 – 3.065)	0.001
T stage	T1	1.000			
	T2	1.684 (1.301 – 2.180)	0.001		
	T3	2.640 (2.012 – 3.465)	0.001		
	T4	4.403 (2.550 – 7.600)	0.001		
N stage	N0	1.000			
	N1	1.652 (1.374 – 1.988)	0.001		
	N2	2.129 (1.658 – 2.735)	0.001		
M stage	M0	1.000			
	M1	2.229 (1.641 – 3.027)	0.001		
TNM stage	IA	1.000		1.000	

	IB	1.244 (0.865 – 1.791)	0.239	0.958 (0.660 – 1.393)	0.824
	IIA	2.229 (1.499 – 3.313)	0.001	1.142 (0.731 – 1.782)	0.560
	IIB	2.382 (1.717 – 3.305)	0.001	1.370 (0.963 – 1.950)	0.080
	III	3.176 (2.204 – 4.578)	0.001	1.654 (1.102 – 2.481)	0.015
	IV	4.310 (2.832 – 6.560)	0.001		
LVI	No	1.000		1.000	
	Yes	1.608 (1.359 – 1.903)	0.001	1.200 (1.000 – 1.439)	0.050
PNI	No	1.000		1.000	
	Yes	1.668 (1.351 – 2.059)	0.001	1.404 (1.116 - 1.768)	0.004
RM	R0	1.00		1.000	
	R1	1.657 (1.382 – 1.986)	0.001	1.344 (1.097 – 1.647)	0.004

BMI, body mass index; CA, carbohydrate antigen; CEA, carcinoembryonic antigen; mGPS, modified Glasgow prognostic score; POPF, postoperative pancreatic fistula; CTx, chemotherapy; CCRTx, concurrent chemoradiation therapy; LVI, lymphovascular invasion; PNI, perineural invasion; RM, resection margin
